# A novel nonsense variant of the *AGXT* identified in a Chinese family: special variant research in the Chinese reference genome

**DOI:** 10.1186/s12882-021-02276-3

**Published:** 2021-03-10

**Authors:** Chang Bao Xu, Xu Dong Zhou, Hong En Xu, Yong Li Zhao, Xing Hua Zhao, Dan Hua Liu, Yong An Tian, Xin Xin Hu, Jing Yuan Guan, Jian Cheng Guo, Wen Xue Tang, Xia Xue

**Affiliations:** 1 The Second Affiliated Hospital of Zhengzhou University, Zhengzhou University, Zhengzhou, China; 2 Academy of Medical Sciences, ZhengZhou University, Zhengzhou, China; 3 Precision Medicine Center of ZhengZhou University, Zhengzhou, China; 4 BGI College, ZhengZhou University, Zhengzhou, China; 5 Henan Institute of Medical and Pharmaceutical Sciences, Zhengzhou University, Zhengzhou, China

**Keywords:** Primary hyperoxaluria type1, *AGXT*, Local genome reference, Whole-exome sequencing, case report

## Abstract

**Background:**

Primary hyperoxaluria(PH)is a rare autosomal recessive genetic disease that contains three subtypes (PH1, PH2 and PH3). Approximately 80% of PH patients has been reported as subtype PH1, this subtype of PH has been related to a higher risk of renal failure at any age. Several genetic studies indicate that the variants in gene *AGXT* are responsible for the occurrence of PH1. However, the population heterogeneity of the variants in *AGXT* makes the genetic diagnosis of PH1 more challenging as it is hard to locate each specific variant. It is valuable to have a complete spectrum of *AGXT* variants from different population for early diagnosis and clinical treatments of PH1.

**Case presentation:**

In this study, We performed high-throughput sequencing and genetic analysis of a 6-year-old male PH1 patient from a Chinese family. Two variants (c.346G > A: p.Gly116Arg; c.864G > A: p.Trp288X) of the gene *AGXT* were identified. We found a nonsense variant (c.864G > A: p.Trp288X) that comes from the proband’s mother and has never been reported previously. The other missense variant (c.346G > A: p.Gly116Arg) was inherited from his father and has been found previously in a domain of aminotransferase, which plays an important role in the function of AGT protein. Furthermore, we searched 110 pathogenic variants of *AGXT* that have been reported worldwide in healthy local Chinese population, none of these pathogenic variants was detected in the local genomes.

**Conclusions:**

Our research provides an important diagnosis basis for PH1 on the genetic level by updating the genotype of PH1 and also develops a better understanding of the variants in *AGXT* by broadening the variation database of *AGXT* according to the Chinese reference genome.

**Supplementary Information:**

The online version contains supplementary material available at 10.1186/s12882-021-02276-3.

## Background

Primary hyperoxaluria type1(PH1, OMIM:259900) is a rare glyoxylate metabolism disorder caused by the functional deficiency of alanine glyoxylate aminotransferase (AGT). AGT is a liver-specific enzyme and its deficiency can damage the glyoxylate metabolism in peroxisomes of hepatocytes by causing oxalate-overproduction [1]. Compare with PH2 and PH3, PH1 has a higher incidence and risk of leading to renal failure [2, 3]. Clinically, PH1 has been reported as a highly heterogeneous disorder with various clinical performances that range from asymptomatic to renal failure [1]. Thus, it is difficult to confirm PH1 at the early stage, as several have not been conclusively linked to symptomatic or asymptomatic conditions. PH1 could be fatal, especially for young children, about half of the PH1 patients with early onset will have end-stage renal disease (ESRD) before adulthood [1, 4, 5]. Therefore, it is vital to find an accurate approach to confirm PH1 at the early stage in order to provide timely and appropriate treatments. PH1 has significant genetic heterogeneity and phenotypic diversity [6], and gene *AGXT* has been identified as the key gene associated with the onset of PH1 [2, 7, 8]. A complete pathogenic variant spectrum of *AGXT* from different populations would provide a valuable resource for diagnosing PH1 at the genetic level by exposing potential genetic markers for the PH1 subtype of this diease.

*AGXT* (OMIM:604285), maps to chromosome 2q37.1, is about 10 kb with 11 exons that encode the AGT protein [9]. The variants in gene *AGXT* could be found in more than 99% of PH1 patients [7, 10]. To date, at least 190 different pathogenic variants have been reported in gene *AGXT*, over 55% of these variants are missense that could result in aberrant gene products [7–10]. Compare with missense variants in *AGXT*, fewer nonsense variants have been described worldwide, and the impacts of nonsense variants on the AGT protein functioning are uncertain [11]. Benefiting from the rapid development of Next Generation Sequencing (NGS) technology, more *AGXT* variants have been recently identified in the Chinese population [12–14].

In the present report, a six year old boy with confirmed PH1was treated conservatively for five months. Two variant points in gene *AGXT* were identified in his family by Whole Exon Sequencing (WES), including a novel nonsense variant from his mother and a missense variant from his father [6, 15, 16]. To update PH1 genotypes in Chinese population and elucidate the *AGXT* variants spectrum in the local population, we built a local reference genome by collecting genomes from 300 healthy Chinese Han people. By searching110 pathogenic variants of *AGXT* in the local reference genomes, we aim to provide more reliable genetic markers for PH1 early diagnosis and treatment in Chinese population.

## Case presentation

### Clinical data of a young PH1 patient

**Fig. 1:**
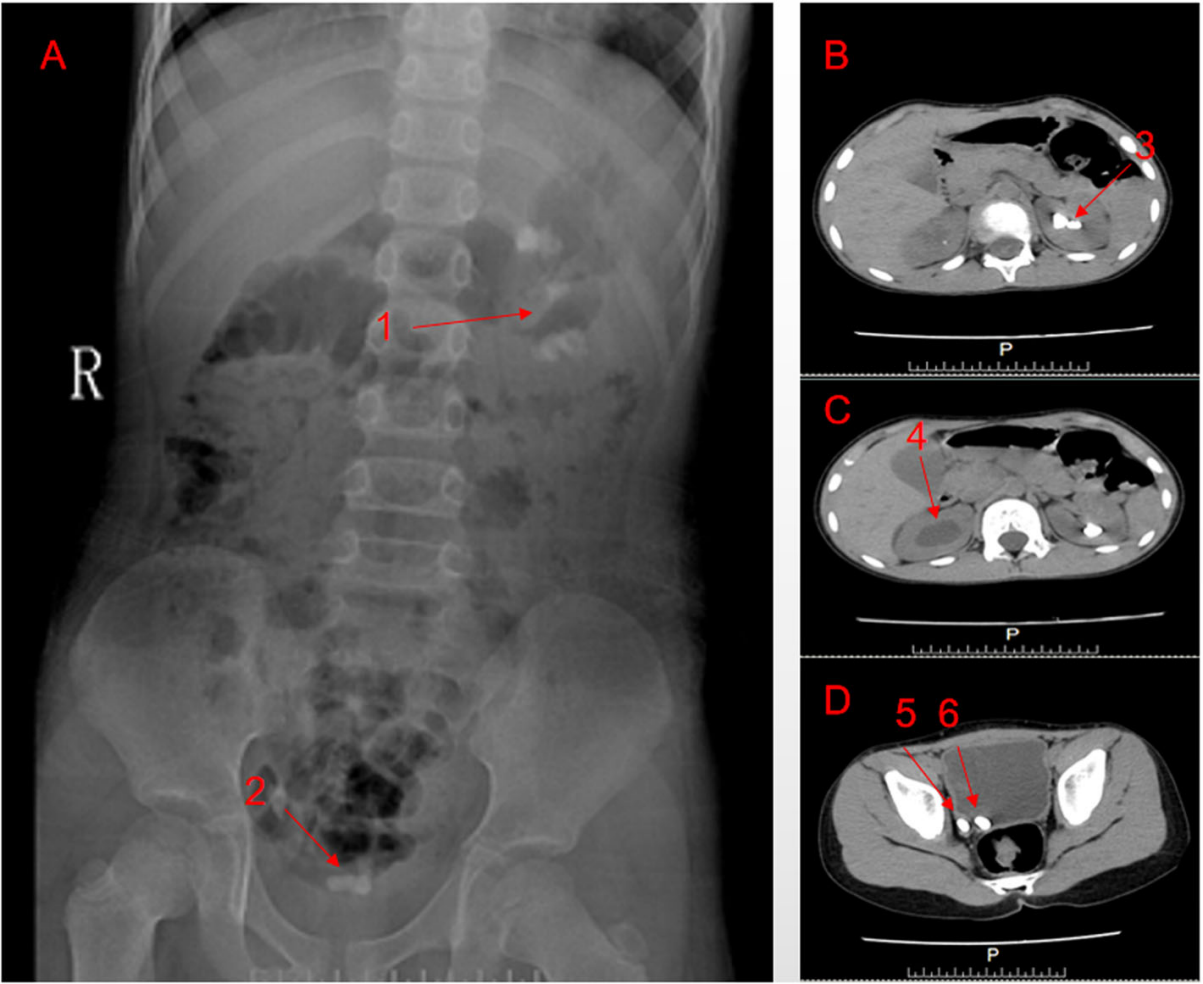
Radiographic findings in patient, the arrows indicate stones. **a**: KUB film, R indicates the right side of the body and the two red arrows indicate the stone detected in the kidney (arrow 1) and the bladder (arrow 2); **b**, **c**, **d** (CT axial image): **b**: the average CT value of arrow 3 is 1395.28HU; **c**: arrow 4 indicates hydronephrosis in the right kidney; **d**: the average CT values of arrows 5 and 6 are 1501.58HU and 1283.38HU respectively

The patient is a 6-year-old Chinese boy (ethnic Han), who was admitted to the hospital due to dysuria and intermittent abdominal pain for the last ten days. A stone was passed spontaneously by the patient seven days before sent to the urological clinic. The boy was born in Henan Province of China (Central China, Han) and was the second child of non-consanguineous parents. The family denied the presence of PH in the family history. The physical examination of the patient was in normal range. The boy has moderate nutrition, normal intelligence, a height of 130 cm, a weight of 24 kg, a blood pressure of 105/77 mmHg. A computerized tomography (CT) scan showed the hydronephrosis in his right kidney, and stones occurred in his left kidney, right ureter and bladder (Fig. 1). The crystals passed by the patient spontaneously were identified as mostly (> 95%) composed of calcium oxalate monohydrate according to the infrared spectroscopy test (Supplementary Fig. 1). From the examination of his renal and liver function, we found the serum creatinine concentration was as low as 2.07 mmol/1.73m^2^/24 h, the concentration of urinary oxalate was 2.44 mmol/1.73m^2^/24 h (Table 1).

**Table 1:** Biochemical features of the patient with primary hyperoxaluria

		Normal range
**Blood tests**		
Renal function and Liver Function		
WBC (× 10^9^/L)	5.92	5–12
Hb (g/L)	133	110–160
PLT (× 10^9^/L)	403	100–400
NEUT (%)	51.5	40–75
LYMPH (%)	40.5	20–50
MONO (%)	5.7	3–10
EO (%)	1.5	0.4–8
BUN (mmol/L)	4.56	3.9–7.1
Cr (μmol/L)	40	44–115
UA (μmol/L)	464	90–350
TBIL (μmol/L)	9.6	0–23
DBIL (μmol/L)	3.4	0–6.8
TP (g/L)	75.7	65–85
GLO (g/L)	28.9	20–40
ALT (U/L)	13	9–50
AST (U/L)	9	15–40
AST/ALT	2.2	0.9–1.2
Electrolyte analysis		
K^+^ (mmol/L)	4.48	3.5–5.3
Na^+^ (mmol/L)	142	137–147
Cl^−^ (mmol/L)	105.4	99–110
Ca^2+^ (mmol/L)	2.52	2.11–2.52
Mg^2+^ (mmol/L)	0.79	0.75–1.02
P (mmol/L)	1.61	0.85–1.51
**Urine tests-24 h**		
24-h urine output (ml)	2200	
K^+^ (mmol/L)	18.0	25–100
Na^+^ (mmol/L)	92.0	130–260
Cl^−^ (mmol/L)	222	170–250
Ca^2+^ (mmol/L)	0.8	2.5–7.5
P (mmol/L)	6.8	12.8–22.4
Mg^2+^ (mmol/L)	2.6	2.1–8.2
BUN (mmol/L)	105	
Cr (μmol/L)	2754	
Oxalate (mmol/1.73m^2^)	2.44	

Due to the high concentration of urinary oxalate and the composition of the stone from the patient, we highly suspected that the subject might suffer PH because of a genetic variant inherited from this family. In order to identify the type of PH for the patient and appropriately conduct further treatment, the Whole Exome Sequencing (WES) was performed on the patient (described below).

**Fig. 2:**
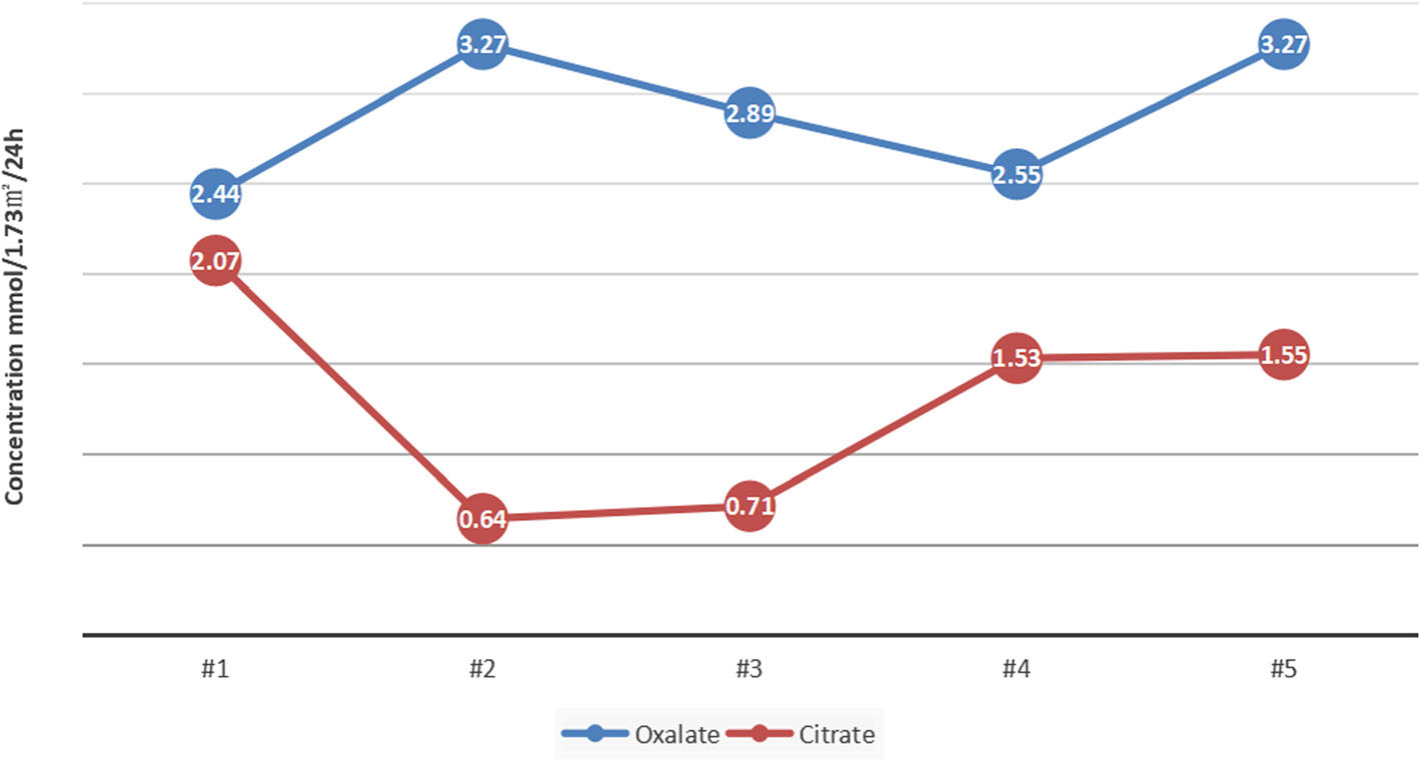
Concentration of Oxalate and Citrate of the patient with primary hyperoxaluria. The patient was reexamined every four weeks. (Data stated in supplementary Table 1)

Based on the four follow-up visits of the patient, the function of his liver and kidney were back to normal and all clinical examinations were within the normal range after the first treatment. Four 2–3 mm new kidney stones were found in the second examination and passed with the aid of physical auxiliary vibration, no obvious stone residue was found afterward. The patient was under a conservative treatment that consisted in a prescription of oral pyridoxine and drinking plenty of fluids (2 ~ 3 L/m^2^ water per day). During this period, the urinary oxalate level decrease but the citrate level increased (Fig. 2).

### WES identifies two variants in the gene *AGXT*

WES was conducted on the patient to identify the specific type of PH that the subject has. We obtained the original data size of 15.74G with 93.2% of bases having Phred quality score of > = 30 (Q30). The mapping rate of sequencing reads to the reference genome was over 99.9%, and the average sequencing depth was 100X with 97% of captured regions that have a minimum depth of 20X. Variant characterization and filtering found two variants in the *AGXT* gene in the proband (supplementary Table 2).

Both variants of *AGXT* found in the proband are heterozygous. The nonsense variant (c.864G > A: p.Trp288X) identified in the proband is on exon 9 and inherited from his mother, which leads to a sense codon changing to a stop codon. The other variant (c.346G > A: p.Gly116Arg) is a missense variant from the father that occurs in a domain of aminotransferase that is essential to the function of the AGT protein.

According to “The Standards and Guidelines for The Interpretation of Sequence Variants” enact by The American College of Medical Genetics and Genomics (ACMG) [17], the nonsense variant (c.864G > A: p.Trp288X) was classified as “likely pathogenic”. This variant has not been reported in the healthy populations from the database of gnomAD and ExAC (PM2). By detecting this variant in the proband’s parents, a likely pathogenic variant was identified at the trans position of the variation site (PM3). Multiple statistical methods predicted that the variant would result in damage to the gene or its products (PP3). Moreover, the clinical phenotype was a promising match for the disease caused by the variant of *AGXT* (PP4). We also classified the missense variant (c.346G > A: p.Gly116Arg) as “likely pathogenic”. This variant has a low frequency (0.00001878) in the healthy population database of genomAD (PM2) and it has been reported as pathogenic/likely pathogenic [15](PMID: 26252291). Additionally, the homozygous variants [6](PMID: 25629080) found at its trans position (PP3) and multiple statistical methods predicted that the variant would result in damage to the gene or its products (PP3). The clinical phenotype was promising with the disease caused by the variant of *AGXT* (PP4) (Supplementary Table 2).

### Confirmation of *AGXT* variants in this Chinese family by sanger sequencing

**Fig. 3:**
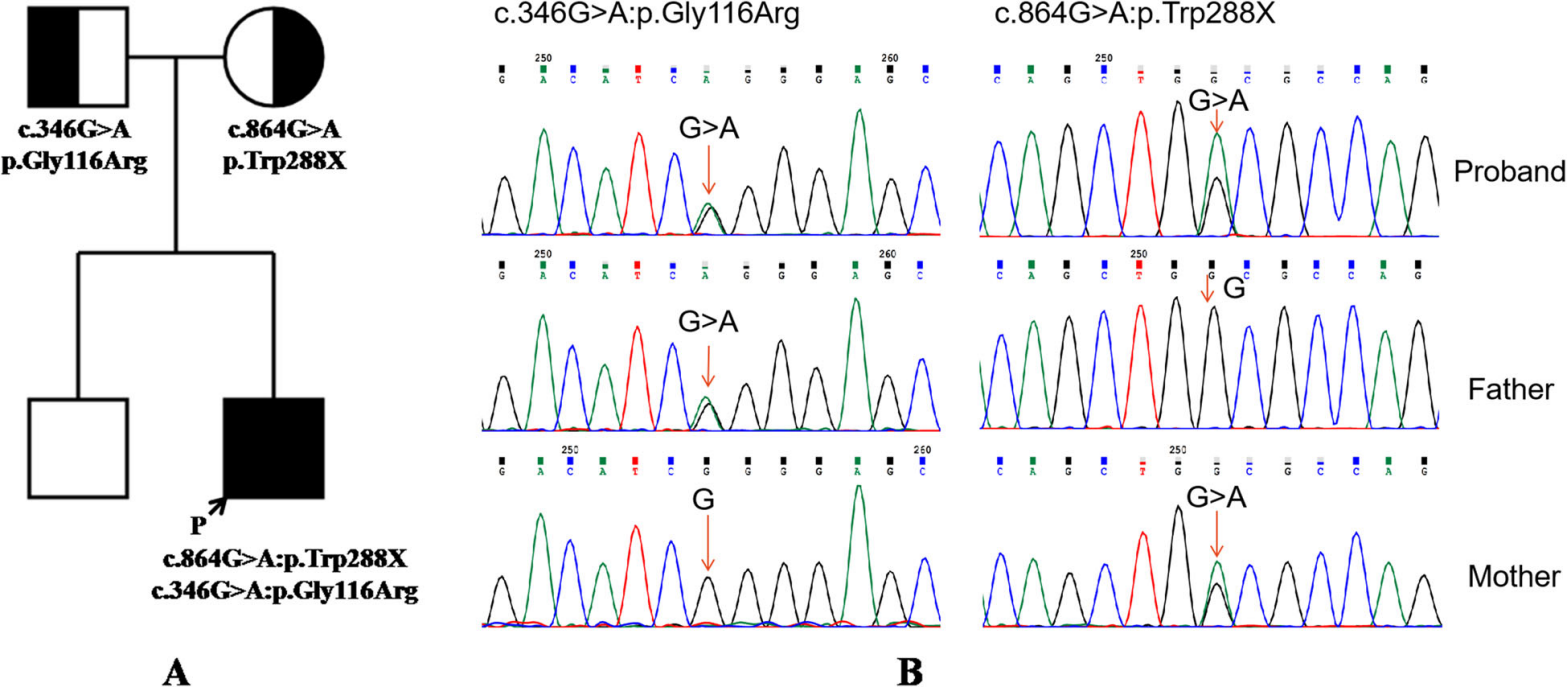
Two *AGXT* variants in a Chinese family of primary hyperoxaluria type 1 (PH1). **a** The pedigree of the Chinese family. **b** Sanger sequencing of the *AGXT* gene variants, c.864G > A:p.Trp288X and c.346G > A:p.Gly116Arg in the family

We designed and synthesized primers flanking the variants of the *AGXT* gene (Supplementary Table 3). Both variants were detected in the proband, while the nonsense variant (c.864G > A:p.Trp288X) was only found in his mother and the missense variant (c.346G > A:p.Gly116Arg) was only found in his father (Fig. 3).

### The frequency of *AGXT* variants in local population

We collected the blood samples from 300 healthy Chinese people locally (Han, Henan Province) and conducted the whole-genome sequencing to establish a local healthy reference genome for studying genetic disorder (Unpublished data). We searched for c.864G > A:p.Trp288X and c.346G > A:p.Gly116Arg of *AGXT* in this local reference genome database and also PGG.SNV dataset (https://www.pggsnv.org/), but both of them were absent from the local reference genome (300 Han people) and PGG.SNV dataset. To test the variant burden in the local population, 110 pathogenic variants (Supplementary Table 4) of gene *AGXT* reported previously (HCMD, the Human Gene Mutation Database, http://www.hgmd.cf.ac.uk/ac/index.php) were searched in this local database. As we expected, none of these rare variants were detected in the local reference genome, which indicates that these rare pathogenic variants in *AGXT* occur at a significantly low frequency in the local population. We also identified the variants in gene *AGXT* of the samples from this local reference genomes and 238 variants found in the healthy local population (Supplementary Table 5).

## Materials and methods

### Whole exome sequencing

The genomic DNA was extracted from the peripheral blood with GenMagBio Genomic DNA Purification kit (GenMagBio, Changzhou, China), and the genomic DNA was fragmented by Bioruptor Pico and quality checked by Agilent Bioanalyzer 4200&2100. We recorded the concentration of the total DNA on the Thermo Scientific NanoDrop One. VAHTS TM Universal DNA Library Prep Kit for Illumina V3 (Vazyme Biotech Co., Ltd., Nanjing, China) protocol was used to build the DNA fragment libraries for each sample. After library preparation, capture and amplification was conducted by SureSelect Clinical Research Exome V2 (Agilent). Illumina 4000 was used to sequence the whole exome. In this study, we detected the whole exon and intron-exon boundaries (50 bp). Human reference genome (GRCh37) was used to do the alignment and analysis.

### Bioinformatics analysis

Trimmomatic-0.39 [18] was used to trim the sequencing adaptors and low quality reads. Alignments of clean reads against the human reference genome (GRCh37 and local) was performed using Burrow Wheeler Aligner (version 0.7.17-r1188) [19]. We used Genome Analysis Toolkit version 4 (GATK4) HaplotypeCaller to call single nucleotide variants and small indels [20], and annotated the variants with Vcfanno [21] using the different annotation databases (1000 Genomes Project database [22], dbSNP [23], Exome Aggregation Consortium (ExAC) [24]). The analysis above was carried out in the bcbio-nextgen (https://bcbio-nextgen.readthedocs.io/en/latest/) pipeline.

### Sanger sequencing

We detected the *AGXT* variant within the patient’s parents’ DNA by Sanger sequencing. Two pairs of primers of c.864G > A and c.346G > A (supplementary Table 3) were designed with NCBI Primer-BLAST and synthesized by ShangYa bio-technology company. PCR products were purified by a PCR purification kit (LifeSciences, Hanzhou, China) and sequenced by a SeqStudio Genetic Analyzer System sequencer (Applied Biosystems).

### Local genome reference construction

300 healthy subjects (Han, China) that aged 18–60 years including 100 males and 200 females were selected based on the physical examination and disease history investigation. All participants signed informed consent forms before blood samples collecting, and the project was approved by the ethics committee of Zhengzhou University.

Genomic DNA was extracted from white blood cells using the GenMagBio Genomic DNA Purification kit (GenMagBio, Changzhou, China). DNA concentration and purity were measured by NanoDrop One (Thermo Fisher Scientific, USA). Purified genomic DNA was segmented and screened with two rounds of beads to obtain the required fragments (~ 400 bp). End-repair of DNA fragmentation, adapter ligation and library amplification were performed following the manufacturer’s protocol for VAHTS TM Universal DNA Library Prep Kit for Illumina V3 (Vazyme Biotech Co., Ltd., Nanjing, China).

### Whole genome sequencing and alignment

The libraries were sequenced by Illumina HiSeq 4000 with pair-end 150 mode (~30X) at the Precision Medicine Center of Zhengzhou University (Zhengzhou, China). Bioinformatics analysis was performed in the framework of bcbio-nextgen (https://github.com/bcbio/ bcbio -nextgen).

### Variants detection in local population

The hardy-Weinberg equilibrium was verified by chi square test. The *p* value of Hardy Weinberg was calculated by Plink, then corrected by fdrtool. The imbalance genetic loci were filtered by 0.05 threshold.

## Discussion and conclusions

In this case, the KUB and CT (Fig. 1) showed hydronephrosis in the patient’s right kidney and stones in his left kidney, right ureter and bladder. At present, the therapies of PH1 is mainly determined by the stage of the PH1 patients are confirmed. The personalized treatment methods are also needed due to various representations of PH1 patients at different ages [8, 25]. It is suggested that the early stage of PH1 should be treated in a relatively conservative manner, for instance, drinking large quantities of water, taking oral pyridoxine, alkaline urine and dietary therapy [25, 26]. In this case, the stones were completely removed by the operation, and conservative treatment has started once PH1 had been confirmed in the subject. For the long run, we aim to reduce urinary oxalate excretion, inhibit stone formation and delay the deterioration of renal function by taking oral citrate, vitamin B6 and drinking water. Once the patient has renal insufficiency, we would conduct liver-kidney combined transplantation in order to avoid the fatal consequences caused by oxalic acid multi-system deposition. The initial urinary oxalate (2.44 mmol/1.73m^2^/24 h) and citrate levels (2.07 mmol/1.73m^2^/24 h) of the subject were set as a baseline, the urinary oxalate and citrate level were evaluated every 4 weeks during treatment (Fig. 2). At the fifth examination, the concentration of urinary oxalate was about 20% decreased, while the creatinine concentration increased to the normal range (1.55 mmol/1.73m^2^/24 h) (Fig. 2 and Supplementary Table 1), besides, the function of his kidney and liver remains normal. Like most diseases, the therapeutic efficacy of PH1 are strongly dependent on its early diagnosis, however, since the clinical representing of PH1 is divergent, it is challenging to diagnose PH1 only with the traditional clinical methods at the earlier stage. Therefore, the genetic examination is demanding, particularly for young PH1 patients .

Three subtypes of PH are all inborn metabolic disorders caused by the specific hepatic enzyme deficiency [6, 25]. The variants in the *AGXT* gene cause PH1 [1, 27], while variants in *GRHPR* result in PH2 [28, 29] and the deficiency in *HOGA1* leads to PH3 [30]. Accordingly, sequencing the whole gene *AGXT* and detecting the variants has become highly recommended for the diagnosis of PH1 at the earlier stage [8, 11, 16]. Although no confirmed variant hot spots in gene *AGXT* have been found [9], more than 190 variants have been reported worldwide in different ethnic populations [4]. In this case, one missense variant (c.346G > A: p.Gly116Arg) and a nonsense variant (c.864G > A:p.Trp288X) of *AGXT* were identified, which only the proband affected by PH1 while the parents and other family members within three generations were asymptomatic. The proband’s mother (c.864G > A:p.Trp288X) and father (c.346G > A: p.Gly116Arg) carries one of the two variants found in him respectively, his older brother is unidentified because of sample missing. Furthermore, neither of the variants from the subject was found in the 300 Chinese reference genomes. The novel nonsense variant on the amino acid position Trp288 was also absent in the GenomAD, ExAc and 1000 Genomes public databases. The subject and his mother both carry this nonsense variant and the subject suffered recurrent nephrolithiasis during the treatment, while his mother’s remained in a good condition. It is of great importance to closely following this patient and his family to develop an understanding of PH1 and this novel variant at a genetic level.

The missense variant on the amino acid position Gly116 has been reported previously and is uncommon compared to the wild-type *AGXT* variants [6, 15, 16]. Chen (2015) and his colleagues detected and reported the variant c.364 > A(p.Gly116Arg) in a 21-year-old man who was diagnosed with kidney failure after 4 months of hemodialysis. The man’s older brother had died earlier due to kidney failure, while his liver function was unremarkable and liver anatomy was normal. This variant could affect the success rate of liver-kidney combined transplantation from PH1 as well. Gly116 missense variant has also been reported in Oman children [16]. Pyridoxine has been known as a metabolic precursor of AGT and could limit oxalate formation PH1 patients efficiently [31, 32]. Two variants in *AGXT* which are c.508G > A (p.Gly170Arg) and c.454 T > A (p.Phe152Ile) have been reported evidently associated with pyridoxine responsiveness [31], which is important for AGT functioning. Moreover, c.121G > A (p.Gly41Arg) has been showed in one case that partially involved in pyridoxine therapy of PH1 [33]. In that study, researchers collected 18 children with PH1 from 4 different non-consanguinity marriage families, and two children younger than 5 years old had this c.346 G > A variant in *AGXT* gene, which is younger than the median age (~ 13 years old) of onset of PH1. In our report, this variant was detected in the proband who is 6-years old which is also at an earlier age compared to the average median age of PH1, and the current treatment works efficiently on preventing the kidney stone from developing. Moreover, since the proband is a heterogeneous variant carrier and his parents carry each variant we found in him, we assume that infecting two pathogenic variants of *AGXT* could result in PH1 representing while infecting one would not in this case. However, whole exon sequencing is efficient for detecting point and indels smaller than 20 bp variants, but not for large fragments indels/duplication of heterogeneous genes, dynamic variants, complex gene rearrangements, GC rich regions, gene regulation regions and intron variants. Therefore, revealing the whole story of *AGXT* variant and PH1 requires further whole genome sequencing and larger patient sample sizes.

We constructed a Chinese local reference genome by collecting 300 healthy people from Henan province and set up a benign variation database of PH1 by comparing the frequency of the pathogenic variants of *AGXT* in the local reference genome to that in the other reference genome database. The variants identified in the subject’s family (c.864G > A:p.Trp288X, c.346G > A: p.Gly116Arg) were not found in the local database, and the known 110 pathogenic variants were not found in the local reference genome database either. It is the first time that we detected the variants of *AGXT* in a Chinese local reference genome and this is significant for Chinese clinical research. However, a larger sampling of a healthy population is still needed. Since the genotype-phenotype correlation in PH1 is controversial in a different population, the local and accurate early diagnosis of PH1 could provide early treatment to local patients and preserve renal function more efficiently. Given the polymorphism of *AGXT*, the diagnosis of PH1 on the genetic level should always be cautious. Currently, various molecular approaches such as substrate reduction therapy [34, 35], pharmacologic chaperones, and gene/cell therapies are developing in cells and PH1 mouse models, which shows promising therapeutic efficacy in animal experiments. However, the further studies on early and accurate diagnosis in PH1 patients are still demanding and would be valuable for finding efficient and appropriate therapeutics to prevent early ESRD and systemic calcium oxalate deposition in different organs with the complications.

## Supplementary Information

**Additional file 1: Supplementary Figure 1.** Infrared spectroscopy of the sediments revealed the crystal as calcium oxalate monohydrate. An automatic infrared spectrum analysis system, LIIR-20 (approved by the Chinese FDA), was used in this study. T%, absorption frequency; WN, wavenumber.

**Additional file 2: Supplementary Table 1.** Concentration of Oxalate and Citrate of the patient with primary hyperoxaluria.

**Additional file 3: Supplementary Table 2**. The pathogenicity classification of the *AGXT* mutations.

**Additional file 4: Supplementary Table 3**. The primers of *AGXT* mutation for Sanger sequencing.

**Additional file 5: Supplementary Table 4.** 110 pathogenic variants of gene* AGXT*.

**Additional file 6: Supplementary Table 5.** 238 variants of gene AGXT in local reference genomes.

## Data Availability

The raw data generated in the current study are available in NODE (http://www.biosino.org/node) with the accession number is OEP000643 URL:http://www.biosino.org/node/project/detail/OEP000643. They are also available from the corresponding author upon reasonable request.

## Abbreviations

PH: Primary hyperoxaluria
PH1: Primary hyperoxaluria type1
AGT: Alanine glyoxylate aminotransferase
ESRD: End-stage renal disease
NGS: Next Generation Sequencing
Q30: Phred quality score of 30
GATK4: Genome Analysis Toolkit version 4
ExAC: Exome Aggregation Consortium
KUB: Kidney, Ureter, Bladder
CT: Computed tomography
WBC: White blood cell
Hb: Haemoglobin
PLT: Platelets
NEUT: Neutrophil count
MONO: Monocyte
EO: Eosinophils
BUN: Blood urea nitrogen
Cr: Creatinine
UA: Uric acid
TBIL: Total bilirubin
DBIL: Direct bilirubin
TP: Total protein
GLO: Seroglobulin
ALT: Alanine aminotransferase
AST: Glutamic oxalacetic transaminase
