# Supplementary material for: A novel nonsense variant of the AGXT identified in a Chinese family: special variant research in the Chinese reference genome
Source: BMC Nephrol. 2021 Mar 10;22:83. doi: 10.1186/s12882-021-02276-3 (PMC7945658; doi:10.1186/s12882-021-02276-3)
Supplement: Supplementary file 1 — Additional file 1: Supplementary Figure 1. Infrared spectroscopy of the sediments revealed the crystal as calcium oxalate monohydrate. An automatic infrared spectrum analysis system, LIIR-20 (approved by the Chinese FDA), was used in this study. T%, absorption frequency; WN, wavenumber. [file 12882_2021_2276_MOESM1_ESM.docx]

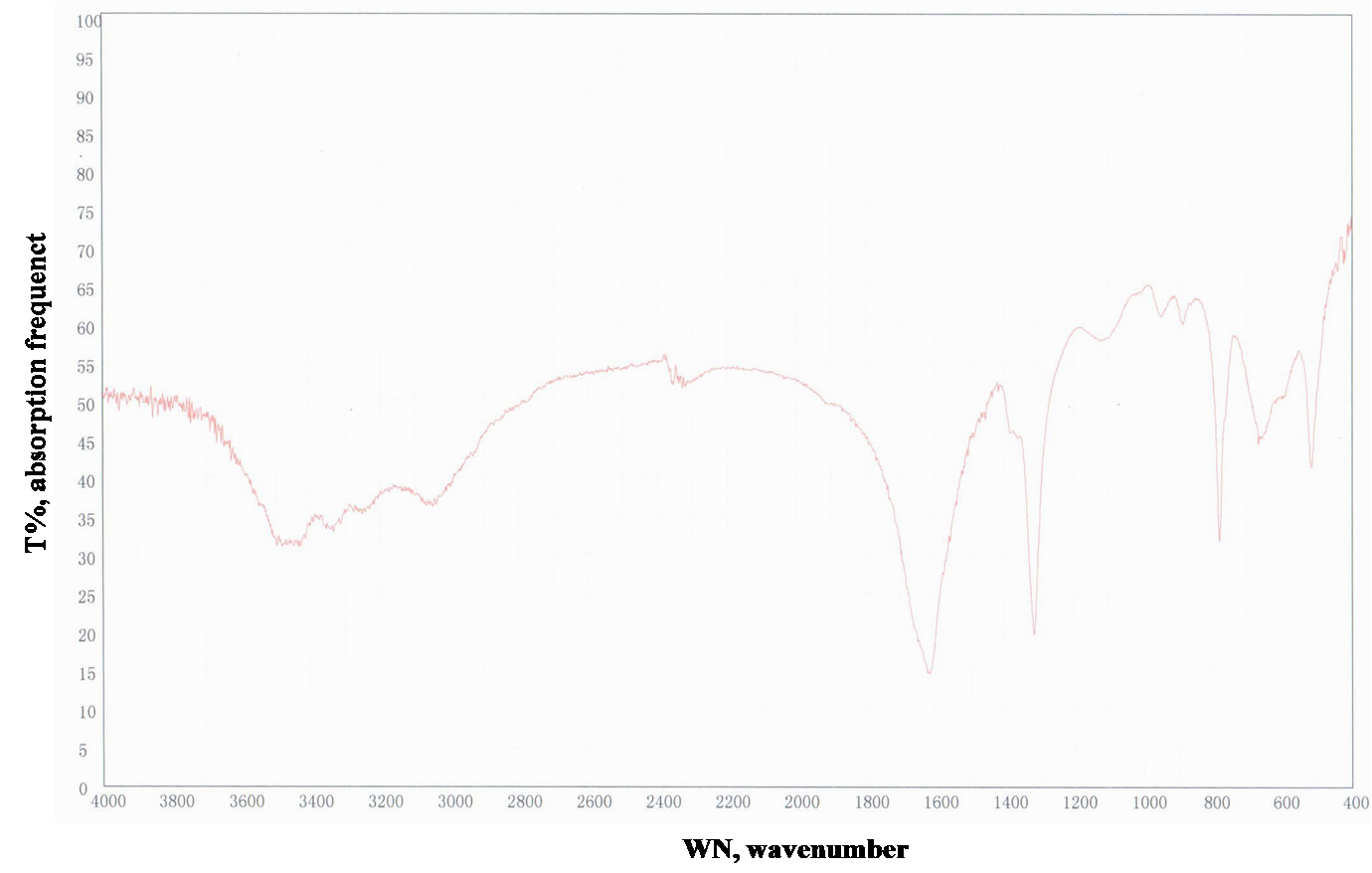


Fig. 1. Infrared spectroscopy of the sediments revealed the crystal as calcium oxalate monohydrate. An automatic infrared spectrum analysis system, LIIR-20 (approved by the Chinese FDA), was used in this study. T%, absorption frequency; WN, wavenumber.
