# Supplementary material for: A novel nonsense variant of the AGXT identified in a Chinese family: special variant research in the Chinese reference genome
Source: BMC Nephrol. 2021 Mar 10;22:83. doi: 10.1186/s12882-021-02276-3 (PMC7945658; doi:10.1186/s12882-021-02276-3)
Supplement: Supplementary file 3 — Additional file 3: Supplementary Table 2. The pathogenicity classification of the AGXT mutations. [file 12882_2021_2276_MOESM3_ESM.docx]

Supplementary Table 2. The pathogenicity classification of the *AGXT* mutations.

| **Transcript** | **Mutation of AGXT** | **Zygosity**  **(Segregation)** | **gnomAD** | **Evidence of pathogenicity** | **ACMG classification** |
| --- | --- | --- | --- | --- | --- |
| **NM_000030.3** | c.864G>A: p.Trp288X | het(p,wt;m,het) | - | PM2,PM3,PP3,PP4 | LP |
| **NM_000030.3** | c.346G>A: p.Gly116Arg | het(p,het;m,wt) | 0.00001878 | PM2,PM3,PP3,PP4 | LP |

LP: likely pathogenic
