# Supplementary material for: A novel nonsense variant of the AGXT identified in a Chinese family: special variant research in the Chinese reference genome
Source: BMC Nephrol. 2021 Mar 10;22:83. doi: 10.1186/s12882-021-02276-3 (PMC7945658; doi:10.1186/s12882-021-02276-3)
Supplement: Supplementary file 4 — Additional file 4: Supplementary Table 3. The primers of AGXT mutation for Sanger sequencing. [file 12882_2021_2276_MOESM4_ESM.docx]

Supplementary Table 3. The primers of *AGXT* mutation for Sanger sequencing

| AGXT Mutation | Forward primer | Reverse primer |
| --- | --- | --- |
| c.864G>A | 5’- CTCTCCACTCTTCTCCCCCA -3’ | 5’- GACTAATCCCTGGCACCGAG -3’ |
| c.346G>A | 5’- ATGTTCCCACCCACAGATCG -3’ | 5’- GCTTCCCTGTAGTTCCGTCC -3’ |
